# Comparative Measurement Accuracy Analysis of an Optical Medium Voltage Transducer Pre- and Post-Lightning Impulse Testing

**DOI:** 10.3390/s26113297

**Published:** 2026-05-22

**Authors:** Grzegorz Fusiek, Pawel Niewczas

**Affiliations:** Department of Electronic and Electrical Engineering, University of Strathclyde, Glasgow G1 1XW, UK; p.niewczas@strath.ac.uk

**Keywords:** fiber Bragg grating, optical voltage sensor, piezoelectric transducer, capacitive voltage divider, power network instrumentation, lightning impulse withstand tests

## Abstract

This paper reports on the performance of an optical voltage transducer (MVT) module after undergoing lightning impulse withstand tests. The device was designed to monitor the output voltage of a dedicated capacitive voltage divider (CVD) to facilitate a voltage sensor dedicated for 132-kV high voltage (HV) networks. Hard piezoelectric transducer (PZT) and fiber Bragg grating (FBG) technologies were combined in the module to serve as a voltage-to-strain-to-wavelength converter. The FBG peak wavelength shifts were calibrated against the input voltage to provide precise measurements of the network voltage. The module was subjected to lightning impulse withstand tests as per the requirements of the IEC 60044-7 and IEC 60060-1 standards, and the impact of the lightning impulses on the performance of the MVT module was evaluated based on the accuracy tests performed before and after the lightning impulse tests. The experimental results demonstrated that the MVT module successfully withstood the lightning impulse tests without any disruptive discharges or voltage collapses. The performance of the module was not affected by the lightning impulse tests within the practical constraints of the reference measuring equipment: its amplitude and phase errors remained within the original limits of ±0.1% and ±0.1° at 80–120% of the rated voltage, and below ±4% and ±2° at 2% of the rated voltage, respectively.

## 1. Introduction

Electricity networks form the backbone of the wide-area power system, enabling the transmission and distribution of electrical energy. The power system grid combines overhead lines, underground cables, transformers, and other equipment to deliver electric energy to the consumers. The AC transmission and distribution networks are operated at various voltage levels ranging from 0.4 kV up to 1 MV [1,2]. The correct operation of the grid can be disturbed by different types of faults, leading to various problems related to the electrical network functionality and stability, including temporary or permanent power outages, failure of the system equipment, or short-term and long-term blackouts [3].

Lightning is one of the leading causes of power outages in the power networks that are triggered by abnormal weather conditions, with lightning strikes being the reason for nearly 10–20% of all faults. It can cause various types of electrical faults, including power surges, equipment damage, and transformer failures. Usually, lightning events cause temporary faults on electrical networks, with less than 20% causing permanent damage [3,4,5]. Electrical faults caused by lightning strikes can affect both residential and commercial customers, as well as transportation systems and other critical infrastructure. While lightning protection systems can help reduce the risk of lightning-related electrical faults, they cannot provide complete protection. In most scenarios, when a lightning event occurs, equipment insulation is at risk of damage due to the substantial surge in line current, which generates significant voltage spikes that can cause flashovers. Mitigation consists of protecting equipment with surge arresters [3] or with sufficient insulation that meets industry standards [6,7]. Lightning-related electrical faults can be costly to repair, especially if they result in the replacement of expensive equipment or the need for rewiring of the existing network infrastructure.

Wide-area monitoring, protection, and control (WAMPAC) systems have been introduced with the aim of enhancing grid performance. These systems play a crucial role in increasing the visibility of the grid, improving response times to network demands and disturbances, and enhancing overall network reliability and security. They are indispensable in preventing blackouts [8,9].

To complement the developing WAMPAC technologies, novel sensor systems enabling multiple, remote, distributed, passive current and voltage measurements over long distances were introduced by the authors. These measurement systems are based on the concept of photonic sensors utilizing fiber Bragg grating (FBG) sensors and piezoelectric transducers. The solutions included optical voltage and current sensors that could be applicable to a wide range of metering and protection applications at low voltage (LV), medium voltage (MV), and high voltage (HV) levels [10,11,12]. For voltage measurements on HV networks, two variants of an optical voltage sensor (OVS) were previously developed for the 132 kV voltage level. The first variant utilized a low-voltage “soft” piezoelectric transducer (LVT) to monitor the output of a bespoke capacitive voltage divider (CVD) having a voltage division ratio (VDR) of 5000 [10]. An alternative design utilized a “hard” piezoelectric (PZT) transducer capable of monitoring a dedicated CVD with a rated 1-kV output voltage [11,12]. Since the “hard” piezoelectric transducers have generally better stability and long-term performance as opposed to the “soft” piezoelectric transducers [13,14,15], the medium voltage transducer (MVT) module demonstrated better linearity, narrower hysteresis, and lower measurement errors than the LVT unit [10,11,12].

However, before the installation on any network, the devices are required to successfully undergo lightning impulse withstand tests according to the safety requirements of the IEC standards. Although LVTs can be protected against damage due to the overvoltage conditions using electronic components such as resistors and transient voltage suppressing (TVS) diodes, this can be a challenging task for piezoelectric transducers that directly sense the multi-kV-level voltages [10,11,12]. As shown in [16], the first MVT prototypes based on the direct MV measurement approach were not capable of withstanding the required lightning tests, and additional bespoke lightning protection components had to be implemented [16].

To overcome the lightning protection problems but exploit the hard PZT performance benefits, an MVT module to work in tandem with a bespoke CVD was proposed by the authors as reported in [11,12]. The proposed MVT was specifically developed for the 132-kV networks, but it could be adapted to other network voltage levels by providing a suitable CVD. The design of the MVT was evaluated theoretically and optimized by means of finite element analysis (FEA) to reduce the electric field and stress in the material during the lightning impulse events to safe levels, so that the transducer would not be damaged [11]. The initial characterization of the first MVT module prototype was carried out to assess its measurement capabilities, meeting the requirements of 0.1 metering and 1P protection classes specified by the IEC 60044-7 and IEC 61869-11 standards [1,6,7,12].

This paper presents follow-on research on the newly developed MVT module [12] and investigates the impact of lightning impulse withstand tests on its measurement accuracy. The module was subjected to lightning impulse withstand testing in accordance with the requirements of IEC 60044-7 and IEC 60060-1 [6,17]. Its performance was evaluated through accuracy tests conducted before and after the impulse testing, complemented by a purely relative stability analysis. The results demonstrate that the new MVT design withstood the specified lightning impulse tests without measurable degradation in accuracy, thereby demonstrating the robustness of the design under standardized impulse conditions.

## 2. Materials and Methods

### 2.1. Voltage Measurement and Voltage Withstand Requirements

In a three-phase 132-kV network system, the single-phase rated voltage is 76.2 kV (107.8 kV peak), and a rated voltage factor of 1.2 applies to measurements between phase and earth continuously. A voltage transformer (or a voltage sensor) connected to such a system is expected to withstand the rated power frequency voltage of 275 kV for 60 s. The device should also withstand 15 positive and 15 negative impulses of the rated lightning impulse voltage of 650 kV, as specified by the IEC 60044 and IEC 61869 standards [6,7].

### 2.2. Voltage Measurement Accuracy Requirements

Since the sensor under development aims at being compliant with the protection and metering classes, its voltage measurement errors at the rated frequency (50 Hz) must be below the limits specified by the IEC standards [6,7,12]. For metering class, the voltage (ratio) and phase errors are determined at the rated frequency and at voltages between 80% and 120% of the rated voltage [6,7]. These requirements are relaxed for the protection class, although they should be determined for voltages ranging from 2% to 120% of the nominal voltage. Additionally, the voltage measurement accuracy at the rated voltage and at frequencies equal to 96% and 102% of the rated frequency for protection class devices, and at frequencies equal to 99% and 101% of the rated frequency for metering class devices, must remain within the stated limits [6,7].

### 2.3. HV-to-MV Voltage Divider

**Figure 1:**
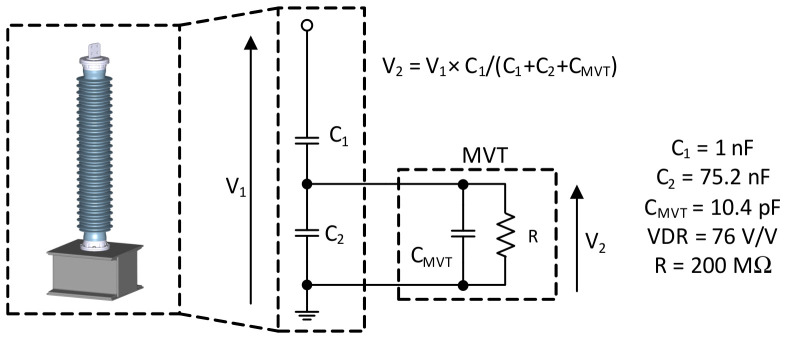
A diagram of an HV-to-MV voltage divider [12].

As mentioned earlier, the MVT module under development was designed to monitor the output of a bespoke high voltage capacitive voltage divider (HV CVD) with a rated output voltage of 1 kV (1.41 kV peak) [11,12]. To bring a single-phase voltage of a 132-kV network system down from 76 kV to 1 kV, the CVD voltage division ratio (VDR) of 76 was proposed. With a given MVT capacitance of 10.4 pF and considering the HV CVD configuration depicted in Figure 1, capacitors C_1_ and C_2_ should have the practical values of 1 nF and 75.2 nF, respectively [11,12].

**Figure 2:**
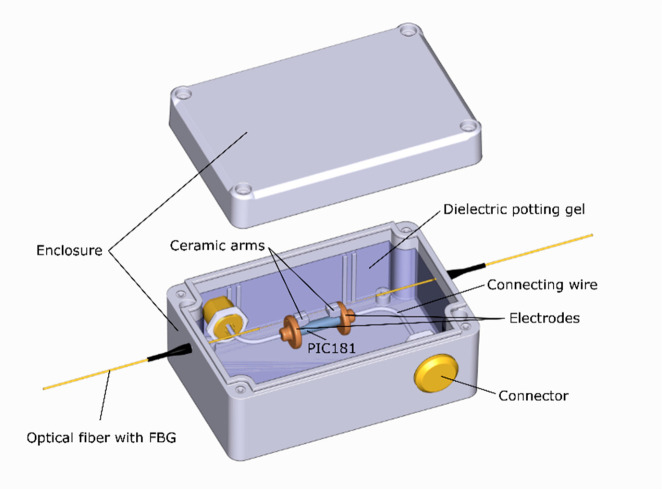
A medium voltage transducer packaged into an ABS enclosure and potted with a dielectric gel [12].

The CVD is expected to be housed in a single HV composite insulator provided by an external supplier, as presented in [10,12]. The assembly would be equipped with an isolated medium voltage terminal to provide access to the medium voltage output inside a suitable enclosure accommodating the MVT module (a fully developed variant of that shown in Figure 2). It is presumed that the CVD will meet the power frequency and lightning impulse withstand voltage standards, as well as the partial discharge limits specified in the appropriate IEC standards [10,11,12]. The design of the bespoke capacitive voltage divider is regarded as a topic for future research and is beyond the scope of this paper.

### 2.4. Medium Voltage Transducer

The detailed design of the medium voltage transducer under development was previously presented by the authors in [11,12]. This combines fiber Bragg grating (FBG) and piezoelectric technologies. In the proposed configuration, an FBG sensor was suspended between two ceramic arms and affixed to two metallic electrodes that bridged a single cylindrical block of PIC181 piezoelectric material from Physik Instrumente (PI), Karlsruhe, Germany [11,12,18]. The piezoelectric element, with dimensions measuring 20 mm in length and 5 mm in diameter, along with the ceramic arms, was securely attached to the electrodes using thin layers of conductive epoxy. Simultaneously, the fiber was attached to the arms using a UV epoxy [12]. Strain, which was proportionate to the input voltage and transferred to the FBG by the piezoelectric component, was calibrated in terms of the measured voltage. The construction of the MVT was designed to provide theoretical twofold strain amplification. This sensor is well-suited for remote interrogation, and by monitoring the instantaneous shifts in the FBG peak wavelength, it allows for the reconstruction of the input voltage. Additionally, tracking the average wavelength permits the determination of the local sensor temperature, which can be employed for temperature compensation of the sensor voltage readings. To prevent the depolarization and permanent damage of the piezoelectric component, its nominal voltage was set at 1 kV. The material stress limits, both in compression and tension, are 100 MPa and 10 MPa, respectively, while the allowable electric field limit is 2.5 kV/mm, as specified in [11,12]. As it was demonstrated in [11] by FEA models, with a nominal voltage of 1 kV applied to the sensor, the expected electric field within the PZT component is approximately 0.07 kV/mm. Under a power frequency withstand voltage of 5.1 kV, a maximum field of 0.25 kV/mm can be anticipated, and it rises to nearly 0.6 kV/mm when subjected to the lightning impulse waveform with a peak of 8.5 kV. Importantly, both the compressive and tensile stress levels generated in the piezoelectric material during the lightning impulse tests should remain within the allowable limits for the material.

### 2.5. MVT Module

A CAD drawing of the MVT module is shown in Figure 2 [12]. The MVT was sealed inside an ABS (Polyacrylonitrilebutadiene-styrene) enclosure, which was fitted with a polyurethane gasket. It is important to clarify that the ABS enclosure was employed to establish an appropriate setup for the initial evaluation of the prototype device and will undergo modifications for its integration with a dedicated CVD in the future.

The interconnections between the MVT electrodes and the external connectors were established using copper conductors with a diameter of 2 mm. To enhance the distribution of the electric field and diminish the electric field strength surrounding the transducer, the device was encapsulated with a dielectric gel. This gel has a dielectric strength of 23 kV/mm and a dielectric constant of 2.7. The curing process of the gel took place under vacuum conditions to facilitate the removal of gas bubbles [12]. Subsequently, the sensor was calibrated and subjected to testing, as detailed in the following sections.

## 3. Results

### 3.1. Experimental Setup

**Figure 3:**
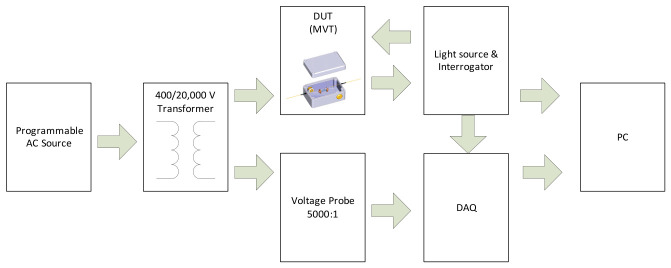
Experimental setup for MVT calibration and testing.

The utilized experimental setup, diagrammed in Figure 3, and procedures were similar to those presented in [12]. The calibration and testing of the MVT module were carried out following a comparison method outlined in IEC 60060-2 [19]. The device under test (DUT) was connected in parallel with a voltage reference system, and the calibration was accomplished by comparing the outputs of both measurement systems, following a methodology similar to that described in [12].

The prototype MVT was calibrated and tested in laboratory conditions at a room temperature of 20 ± 1 °C. The experiments were carried out using a DP04-10K-LVC precision differential voltage probe (CIC Research Co., Ltd., Bangkok, Thailand), having a DC and AC accuracy of 0.1% as a voltage reference [20]. The 0.1% voltage probe (VP) was an active probe with the nominal 10,000:1 voltage division ratio. The device was capable of accurately measuring both DC and AC voltages up to 6 kV and held an ISO17025-accredited calibration certificate for DC and AC gain. The output from the VP was continuously monitored using a specialized PCIe 6363 data acquisition card (DAQ) from National Instruments [21], which was installed in a personal computer (PC).

A step-up transformer (Majestic Transformers, Poole, UK) with a voltage ratio of 400/20,000 V/V was used to provide the test voltage to DUT, while a programmable AC source, Chroma 61512, capable of delivering 18 kVA at 300 V, was employed to power the transformer.

**Figure 4:**
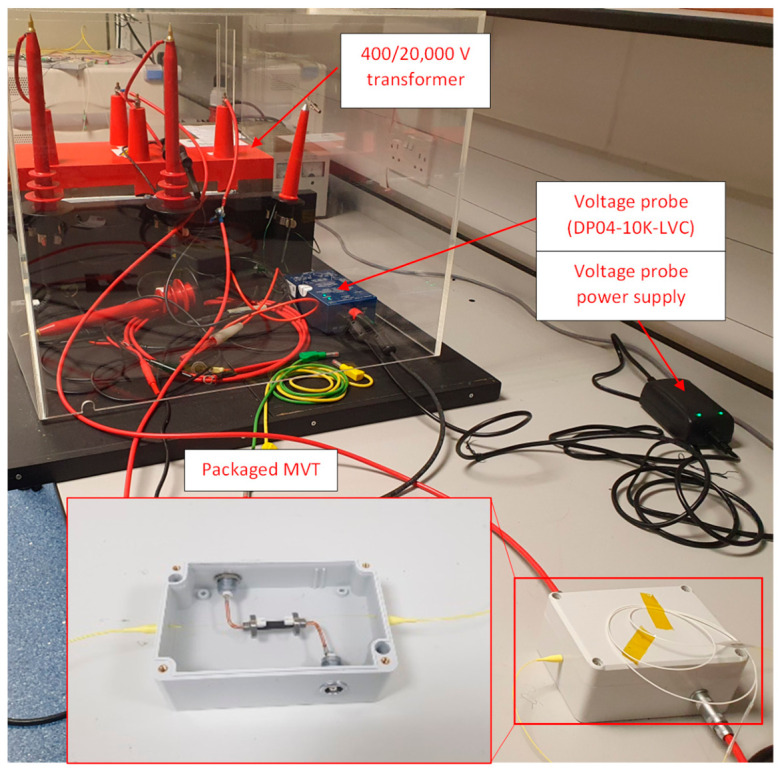
The MVT module connected to a single phase of a 20 kV 3-phase transformer.

A photograph depicting the MVT module linked to a 400/20,000 V transformer, along with the voltage probe during testing, is shown in Figure 4. The MVT module received power from one of the phases of the 20 kV 3-phase transformer.

For the calibration and precision assessment, the MVT sensor was exposed to a broadband light source, and the reflected signals were examined using an FBG interrogator known as I-MON 256 USB (manufactured by Ibsen Photonics, Farum, Denmark, [22]) that was linked to the PC. The optical system was synchronized with the DAQ readings through an internal clock signal generated by the optical sensor interrogator, and this synchronization occurred at a rate of 4 kHz. The measurements obtained from the optical and electrical measurement systems were utilized for calculating the root mean square (RMS) and measurement errors. The errors in voltage amplitude and phase were determined in accordance with the IEC 61869-11 and IEC 60060-2 standards [7,19].

### 3.2. Sensor Characterization and Accuracy Testing

**Figure 5:**
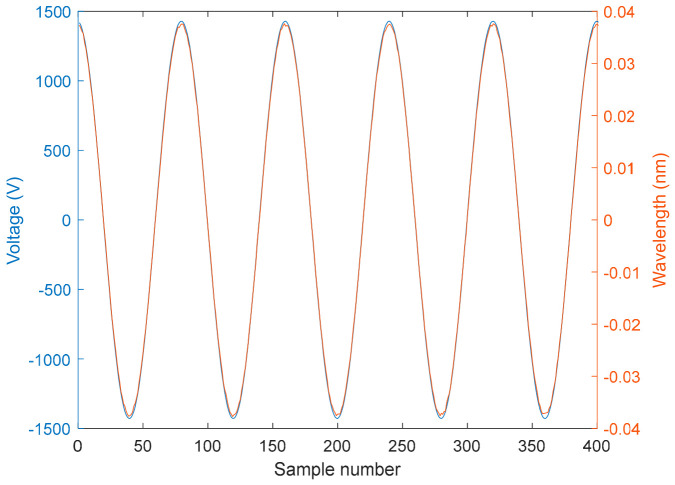
Instantaneous wavelength and reference voltage of the DUT at the nominal voltage.

**Figure 6:**
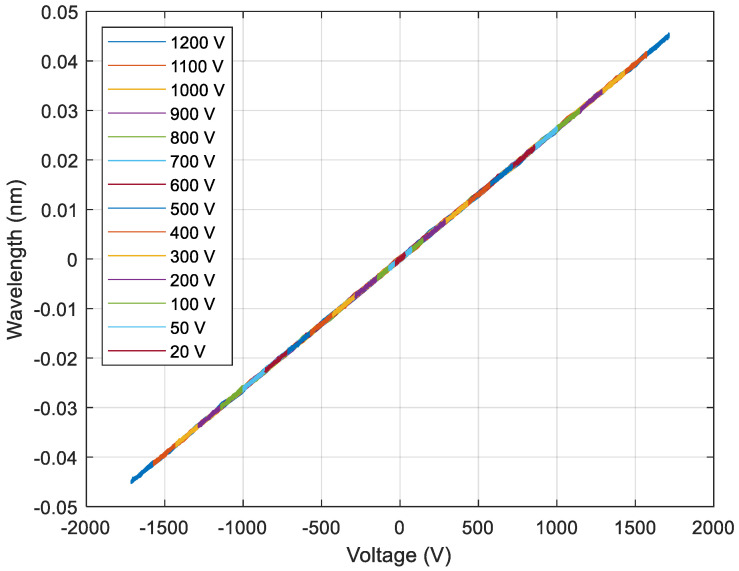
MVT hysteresis for voltages between 2% and 120% of the nominal voltage. The hysteresis between the wavelength and the reference voltage is minimal.

For the purpose of calibrating the MVT, a sinusoidal voltage with a frequency of 50 Hz was applied to the sensor within a range spanning from 2% to 120% of the nominal voltage (1 kV). The voltage levels were set at 2% and 5%, with increments of 10% between 10% and 120% of the nominal voltage. Simultaneously, the optical signals from the sensor and the readings from the voltage probe were recorded at each of these voltage levels. A total of 400 samples, which correspond to 5 periods of 50 Hz signals, were captured at each voltage level. Subsequently, RMS values were computed based on the data collected over 5 periods at each voltage level. The instantaneous reference voltage and wavelength signals at the sensor rated voltage of 1 kV are shown in Figure 5, while the sensor hysteresis behavior across voltage levels ranging from 2% to 120% of the rated voltage is shown in Figure 6. It is evident from the figures that there is no noticeable hysteresis, and the phase difference between the wavelength and the reference voltage is nearly negligible (within the margin of experimental error).

**Figure 7:**
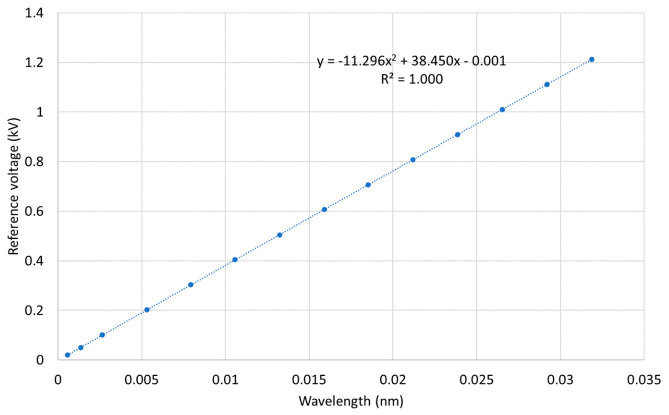
MVT calibration curve before the lightning impulse testing.

The calibration curve for the sensor, as depicted in Figure 7, was generated by fitting a second-order polynomial with a 95% confidence interval to the RMS data acquired during the initial characterization, covering voltage levels ranging from 2% to 120% of the nominal voltage for both reference voltage and wavelength. The resulting quadratic equation was subsequently integrated into the interrogator software. It served as the means to convert the FBG peak wavelength shifts into the corresponding measured voltage levels [12].

As part of the testing procedure aimed at assessing the sensor’s ability to meet protection and metering accuracy standards, 50 Hz voltage waveforms were applied with amplitudes set at 2%, 20%, and within the range of 80% to 120% of the device’s rated voltage. These measurements were conducted in triplicate for thorough evaluation [12].

The amplitude (voltage) error in a steady-state condition is defined according to the following equation [12]:

(1) $$\varepsilon_{u}\left(\%\right)=\frac{U_{p}-U_{rec}}{U_{p}}\times 100$$

where *U_p_* is the RMS value of primary voltage and *U_rec_* is the RMS value of reconstructed voltage.

The phase error is calculated as the difference in phase between the secondary output (reconstructed voltage) phasor and the primary voltage phasor [12]:

(2) $$\varphi_{e}\left({}^{\circ}\right)=\varphi_{s}-\varphi_{p}$$

where *φ_p_* and *φ_s_* are respectively the primary and secondary phase displacements.

**Figure 8:**
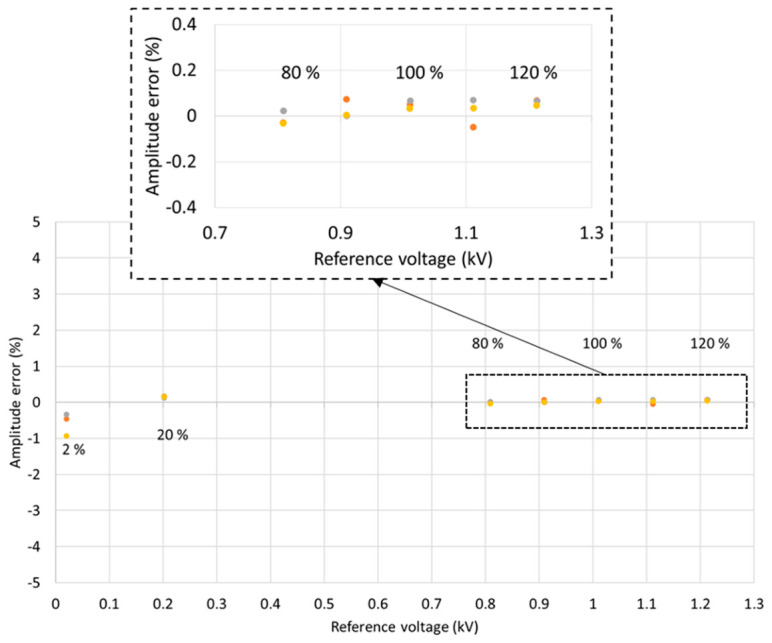
MVT voltage amplitude errors for three consecutive runs before the lightning impulse tests.

**Figure 9:**
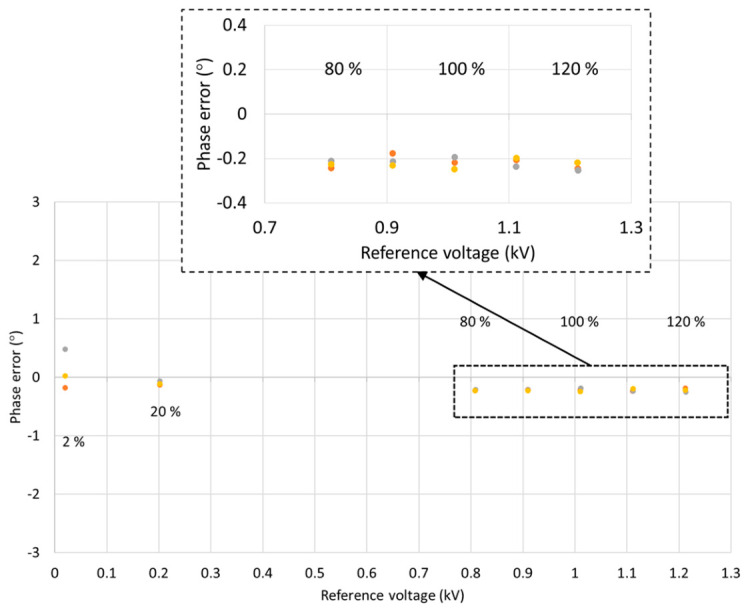
MVT voltage phase errors for three consecutive runs before the lightning impulse tests.

The resultant voltage amplitude and phase errors for the combined three consecutive test runs are presented in Figure 8 and Figure 9, respectively.

**Figure 10:**
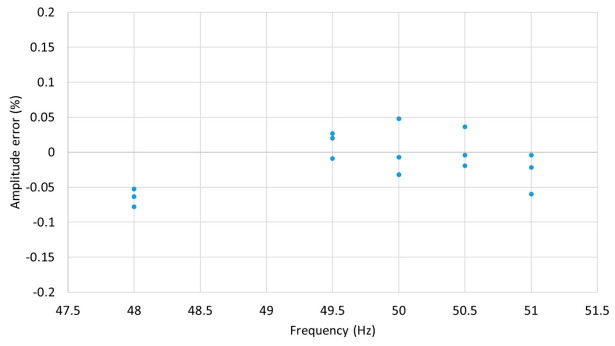
MVT voltage amplitude errors at nominal voltage and frequencies 48 Hz, 49.5 Hz, 50 Hz, 50.5 Hz, 51 Hz, before lightning impulse testing.

**Figure 11:**
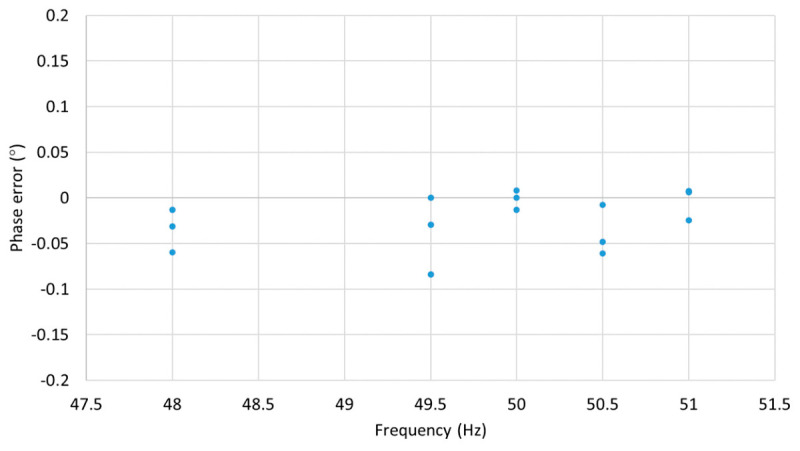
MVT phase errors at nominal voltage and frequencies 48 Hz, 49.5 Hz, 50 Hz, 50.5 Hz, 51 Hz, before lightning impulse testing. The phase errors are shown after removing the −0.2° phase offset.

The subsequent experiment involved assessing the sensor performance under different conditions. This included testing at the rated voltage and frequencies corresponding to 96% (48 Hz) and 102% (51 Hz) of the rated frequency, which are necessary for protection class devices. Additionally, frequencies of 99% (49.5 Hz) and 101% (50.5 Hz) of the rated frequency, required for metering class devices, were examined. The relevant results, in terms of voltage amplitude and phase errors, obtained from three consecutive test runs, are presented in Figure 10 and Figure 11.

As can be seen from the results presented above, the module amplitude and phase errors remained within ±0.1% and ±0.1° at 80–120% of the rated voltage, and below ±4% and ±2° at 2% of the rated voltage, respectively. The phase errors are offset by approximately −0.2°; however, the spread of the error is small, and remains within ±0.1° at voltages near the nominal value. Since the phase offset for the active 0.1% probe is different from the passive 1% probe presented in [12], it is concluded that the offset is introduced by the measurement path between the probe and the DAQ card. It should be noted, however, that a constant phase offset can be corrected by applying a deliberate offset subtraction as stated in IEC 60044-7 and IEC 61869-11 [6,7]. The MVT phase errors when the −0.2° offset has been removed are shown in Figure 11. It should also be noted that the accuracy of the 0.1% probe is stated only for DC and AC measurements, while the phase errors of the probe are not specified or certified by the manufacturer. The long-term stability of the probe also remains unknown.

### 3.3. Lightning Impulse Withstand Tests

In compliance with IEC 60044-7, electronic voltage transformers (EVTs) with the highest equipment voltage, U_m_, of 3.6 kV and above are required to undergo 1.2/50 µs full-wave lightning impulse tests [1]. These impulses are characterized by a front time of 1.2 µs (with a ±30% tolerance) and a time to half-value of 50 µs (with a ±20% tolerance) as defined in IEC 60060-1 [17]. During testing, the DUT is subjected to 15 consecutive positive and 15 consecutive negative impulses and is considered to have passed the tests if no disruptive discharges or flashovers occur, or if there are no more than two flashovers for each polarity. This is confirmed by observing 5 consecutive impulse withstands following the last disruptive discharge. Consequently, the MVT module underwent lightning impulse withstand tests as required by the IEC standards.

As mentioned previously, devices designed for installation in 132 kV networks are mandated by the standard to withstand lightning impulses with a peak voltage of 650 kV [1,6]. At the chosen voltage division ratio of 76 V/V for a dedicated HV CVD, this translates into a peak voltage of 8.5 kV across the MVT module [11,12]. Since there are no facilities at the authors’ laboratory that could be used to carry out lightning impulse tests with a peak voltage of 650 kV, it was decided that the MVT module alone can be tested, assuming the expected proportional peak voltage across the component. Also, the intention of the tests was to verify if the MVT module can survive the relevant voltage levels during the lightning impulse tests before proceeding with the CVD design and fabrication. Consequently, the prototype sensor was subjected to a total of 30 lightning impulses, 15 with positive and 15 with negative polarities, with the required 8.5 kV peak voltage.

**Figure 12:**
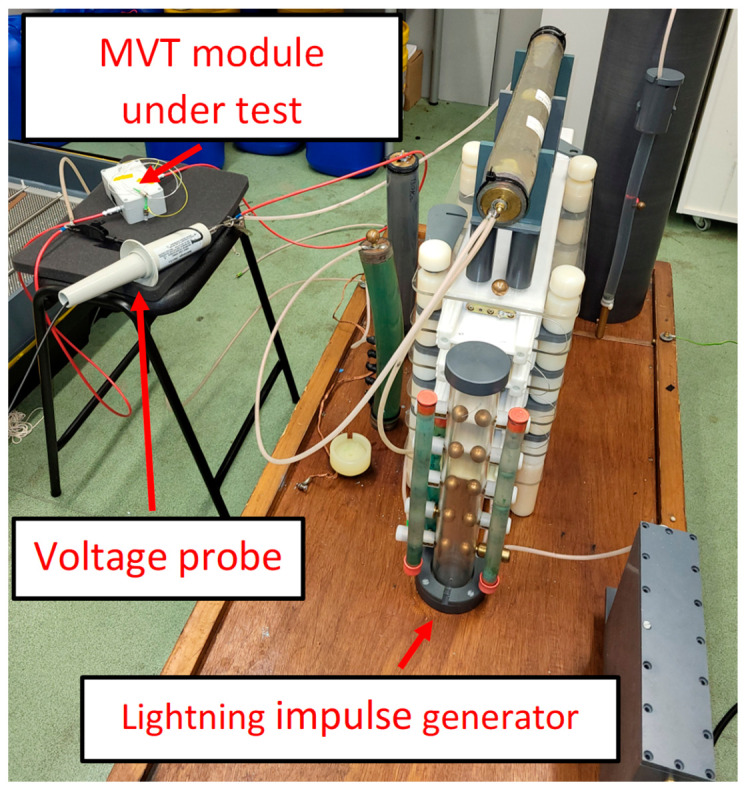
Lightning impulse voltage tests.

To verify the performance of the MVT module regarding its ability to withstand lightning impulses, the tests were conducted by a subcontractor, Samtech Ltd., Glasgow, UK, in accordance with the IEC standards. In these lightning impulse tests, the MVT was connected to a three-stage Marx lightning impulse generator, as illustrated in Figure 12. This generator had the capability to generate the necessary impulses that met the criteria specified by the IEC standards.

**Figure 13:**
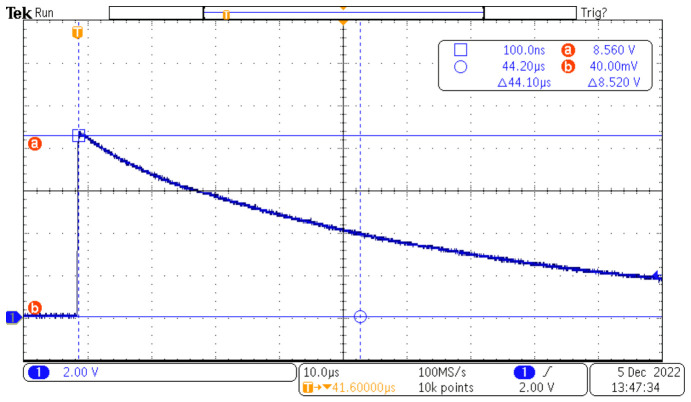
Lightning impulse of positive polarity with a peak of 8.56 kV.

**Figure 14:**
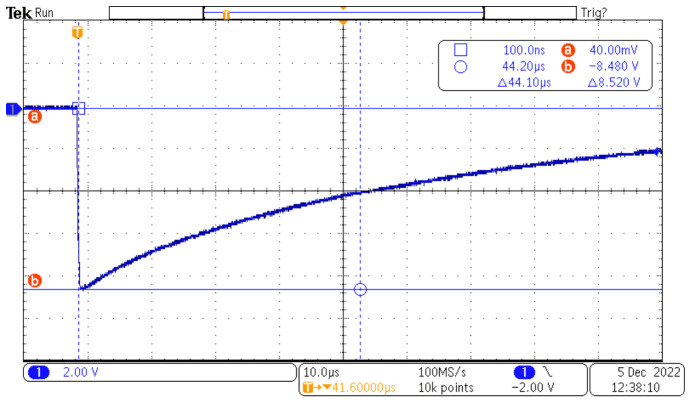
Lightning impulse of negative polarity with a peak of 8.48 kV.

Examples of the 8.5 kV peak lightning impulses with positive and negative polarities that were applied to the prototype MVT sensor during the lightning impulse withstand tests are shown in Figure 13 and Figure 14, respectively.

The MVT module successfully endured all the impulses without any disruptive discharges or voltage collapses.

### 3.4. Accuracy Retesting After Lightning Impulse Tests

After the lightning impulse tests, the MVT calibration and accuracy tests were repeated with the same experimental and data analysis procedures as described in Section 3.2. The reason for recalibration of the sensor after the lightning tests was that the tests were carried out 4 months later than the original calibration of the sensor, and as mentioned in Section 3.2, the long-term stability of the voltage probe cannot be guaranteed. Another important aspect to notice is that the 0.1% probe requires adjustment of the internal op-amp voltage offsets every time it is switched on. The probe also requires some initial time allowed for warming up the probe. Because the voltage offset adjustment is not analog, this may cause some inaccuracies in the measurements, which are especially important when compared to the measurements obtained at different times. The results of the recalibration and accuracy retesting are presented below.

**Figure 15:**
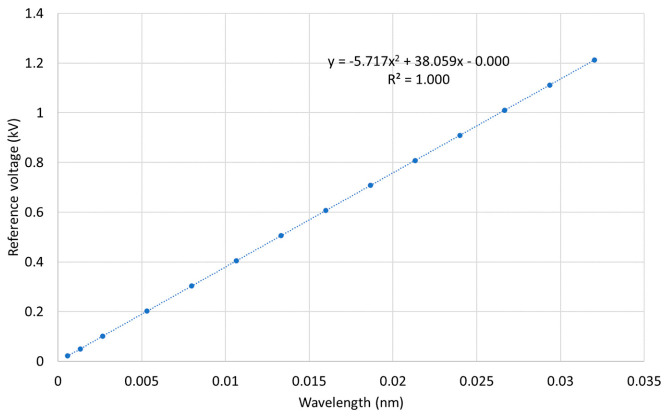
MVT recalibration curve after the lightning impulse testing.

**Figure 16:**
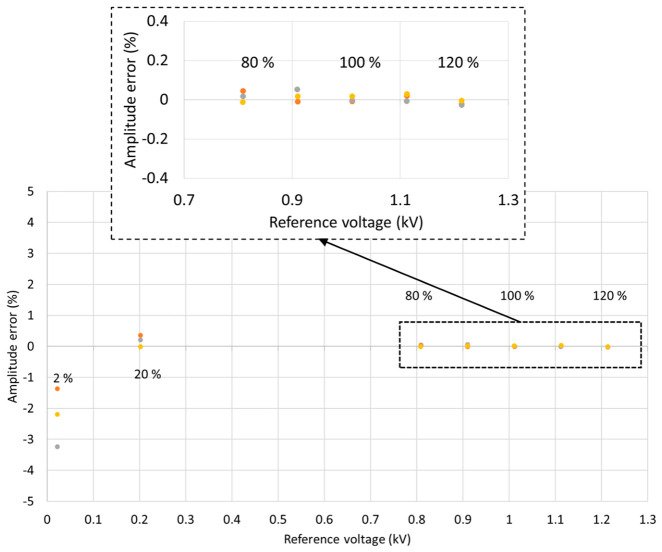
MVT voltage amplitude errors for three consecutive runs after the lightning impulse tests.

**Figure 17:**
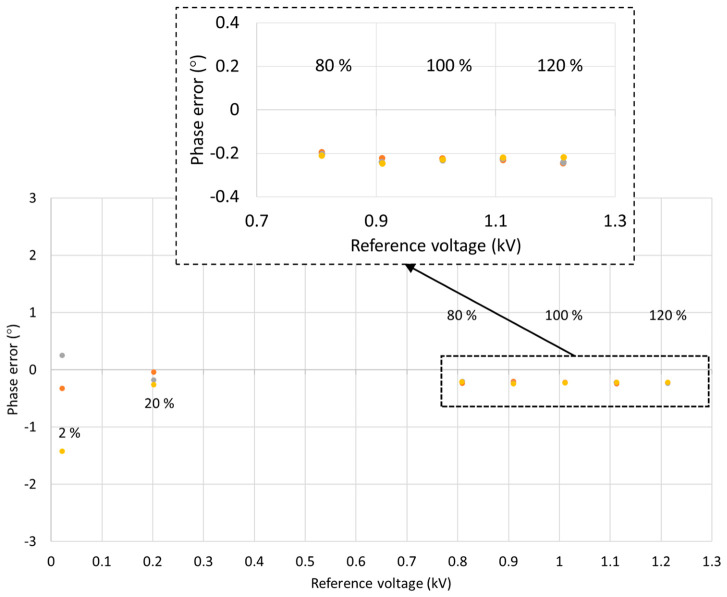
MVT voltage phase errors for three consecutive runs after the lightning impulse tests.

The MVT calibration curve is shown in Figure 15. The amplitude and phase errors are shown in Figure 16 and Figure 17.

The assessment of the sensor performance within the practical constraints of the reference measuring equipment was conducted as previously described in Section 3.2. The resultant voltage amplitude and phase errors for the combined three consecutive test runs are shown in Figure 16 and Figure 17, respectively.

**Figure 18:**
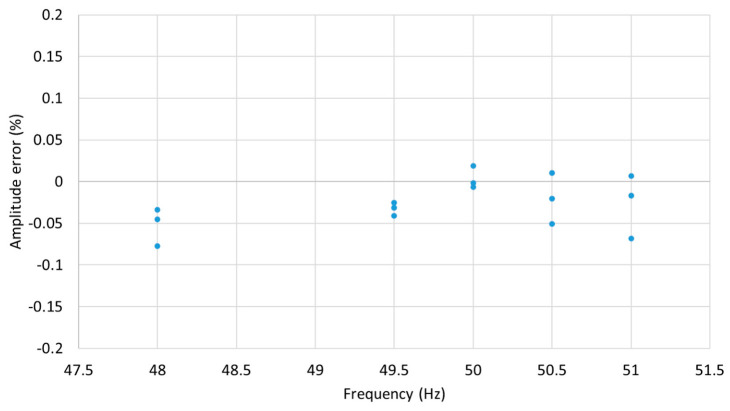
MVT voltage amplitude errors at nominal voltage and frequencies 48 Hz, 49.5 Hz, 50 Hz, 50.5 Hz, 51 Hz, post-lightning impulse testing.

**Figure 19:**
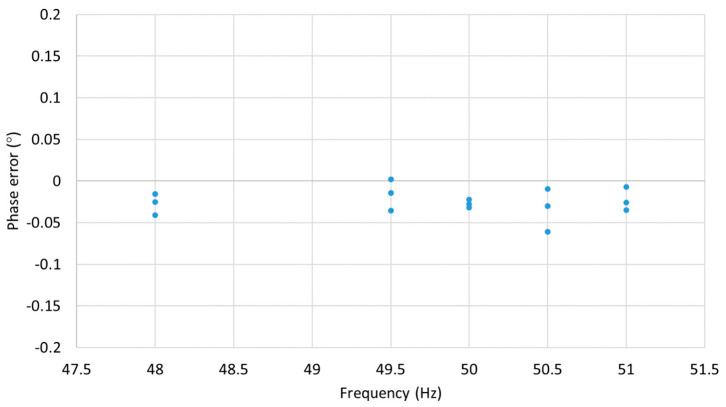
MVT phase errors at nominal voltage and frequencies 48 Hz, 49.5 Hz, 50 Hz, 50.5 Hz, 51 Hz. The phase errors are shown after removing the −0.2° phase offset, post-lightning impulse testing.

The subsequent experiment relied on testing the sensor’s performance at the rated voltage and at frequencies between 96% and 102% of the rated frequency, as previously. The resultant voltage amplitude and phase errors for three consecutive runs are presented in Figure 18 and Figure 19.

As can be seen from the results presented above, the amplitude errors remained within ±0.1% and ±0.1° at 80–120% of the rated voltage, and below ±4% and ±2° at 2% of the rated voltage, respectively. The phase errors spread within the ±0.1° when a constant offset of −0.2° is removed, as discussed in Section 3.2, and as can be seen in Figure 19.

## 4. Discussion

As can be seen from the results presented in Section 3, the MVT voltage and phase errors were within ±0.1% and ±0.1° at 80–120% of the rated voltage, and below ±4% and ±2° at 2% of the rated voltage, for the calibration and accuracy tests carried out before and after the lightning impulse tests required by the IEC 60044-7, IEC 61869-11 and IEC 60060-1 standards. The MVT successfully survived the required train of lightning impulses without any disruptive discharges or voltage collapses. The accuracy tests repeated after the lightning tests showed that the MVT performance had not deteriorated. The 4-month separation between the experiments indicated encouraging long-term stability of the MVT module.

As detailed in Section 3 and discussed in [12], the MVT module exhibits minimal hysteresis and an almost negligible phase displacement between input and output signals. The near absence of hysteresis in the MVT’s response implies that there is no need for extensive signal processing tasks on the interrogator to compensate for hysteresis. By employing solely amplitude scaling, significant signal processing and computational resources can be conserved in the interrogator hardware, which becomes particularly significant when simultaneously interrogating a larger number of sensors. This is a significant benefit of using hard piezoelectric materials for the construction of the MVT module in comparison to the previous incarnation of the HV OVS, which utilized soft piezoelectric materials that required hysteresis compensation to limit the amplitude and phase errors so that the same measurement accuracy could be met.

When comparing the calibration curve obtained during the original sensor calibration and the one resulting from the measurements obtained 4 months later, after the lightning impulse tests, a minimal change in the sensor response over time can be observed. As can be seen from the results presented, the linearity of the MVT response to voltage remained almost unchanged, and the module performance was not affected by the lightning impulse tests within the practical constraints of the reference measuring equipment, as its amplitude and phase errors remained within the limits of ±0.1% and ±0.1° for voltages between 80 and 120% of the nominal, and below ±4% and ±2° at 2% of the rated voltage, respectively. It should be noted, however, that the 0.1% probe is an active probe characterized by a low output impedance, and, therefore, the introduced phase offset due to the measurement path between the probe and the DAQ remained similar after 4 months. The phase errors spread was small, and when the constant phase offset of −0.2° was corrected for, as permitted by IEC 60044-7 and IEC 61869-11 [6,7], the phase error spread remained below ±0.1° as indicated above.

A quantitative uncertainty analysis was performed to assess the reliability of the reported accuracy measurements. Verification of a class 0.1 device ideally requires a reference system uncertainty at least four times lower than the specified class limit. In the present study, the most accurate available reference probe provided a specified AC gain uncertainty corresponding to 0.1% accuracy (0.011 mV RMS, 2σ).

The uncertainty budget was estimated based on equipment specifications and established uncertainty evaluation procedures [23,24,25,26,27,28].

When considering Type B uncertainties, relying on the used equipment specifications provided by the manufacturers, the DAQ card’s absolute accuracy of 0.19 mV (at the selected configuration) resulted in a standard voltage uncertainty of 0.063 mV [21]. As the DAQ acquisition was synchronized with the interrogator clock, the phase uncertainty contribution due to sampling jitter (50 ns at 4 kS/s for a 50 Hz sinusoid) was estimated to be 0.0009°, which is negligible.

The combined wavelength measurement uncertainty of the interrogator, arising from wavelength fit resolution (<0.5 pm standard deviation) and repeatability (3 pm peak-to-peak across polarization states, 1.5 pm half-width uniform distribution) as specified by the manufacturer [22], was estimated to be approximately 1 pm. However, from the analysis of the captured instantaneous optical signals, such as the one shown in Figure 5, the noise floor was 0.2 pm, which indicates that the polarization-induced effects are negligibly small in comparison to the maximum values specified by the manufacturer. With an MVT sensitivity of approximately 27 pm/kV, this corresponds to a standard voltage uncertainty of 7.4 V for the sensor–interrogator chain. Thermal drift was neglected due to temperature-controlled laboratory conditions. Consequently, the resulting combined standard uncertainty in voltage measurement, including contributions from the reference probe, DAQ system, and sensor–interrogator chain, was estimated to be 0.7% at a nominal voltage of 1 kV.

When considering Type A uncertainties arising from the MVT RMS characteristic during the consecutive five runs, the combined uncertainty in the reference voltage and the optical signal was equal to 13 µV and 0.0038 pm, respectively, which translates into a combined standard uncertainty of 0.02% at 1 kV primary voltage.

The experimental results demonstrate a clear distinction between the system’s absolute accuracy (Type B) and its measurement precision (Type A). While the Type B uncertainty is dominated by the fixed noise floor of the optical interrogator and the DAQ card, the Type A uncertainty represents the true repeatability of the measurement process. By calculating the RMS values from 400 samples per signal over five independent runs, the stochastic (random) noise was effectively suppressed through statistical averaging. The low Type A uncertainty proves that the measurement system is stable enough to detect minute changes in the DUT. It confirms that the observed sensor response is a physical phenomenon rather than an artifact of random instrumentation noise. The fact that the Type A uncertainty is 35 times smaller than the Type B limits confirms that the experimental procedure, specifically the synchronization between the high-voltage probe and the optical interrogator, is sufficiently robust. In conclusion, although the absolute uncertainty is constrained by the hardware’s baseline noise floor (Type B), the superior repeatability (Type A) provides the necessary resolution to characterize the optical sensor’s performance with high confidence.

While the system’s absolute accuracy results in a Test Uncertainty Ratio (TUR) below the 4:1 threshold, typically required for commercial certification, the system’s short-time repeatability remains sufficient for research-grade performance evaluation and for assessing the impact of lightning impulses on measurement integrity. However, as the absolute phase accuracy of the reference probe is not specified, formal verification of compliance with the protection and metering classes cannot be fully confirmed.

## 5. Conclusions

The construction of an optical medium voltage transducer (MVT) module, and the results of its calibration and accuracy testing in accordance with the requirements of the IEC 60044-7 and IEC 61869-11 standards, have been presented in this paper. The results of lightning impulse tests, carried out according to the IEC 60060-1 standard, and their impact on the MVT module performance have also been presented and analyzed.

The outcomes of the accuracy tests indicate that the MVT module shows the potential to comply with the accuracy requirements stipulated in the IEC 60044-7 and IEC 61869-11 standards for 0.1 metering and 1P protection devices, as well as multipurpose devices. However, as the absolute phase accuracy of the reference probe is not specified, formal verification of compliance with the protection and metering classes cannot be fully confirmed.

In addition to the accuracy assessments, the MVT module also underwent lightning impulse withstand tests, where it successfully withstood the prescribed sequence of impulses without experiencing any disruptive discharges or voltage collapses. The accuracy tests repeated after the lightning tests showed that the MVT performance was not deteriorated, and the resultant small changes in the calibration and accuracy results can likely be attributed to the inaccuracy of the used voltage probes and the available experimental setup rather than the MVT module instability. Although the MVT module aims at monitoring the output of a dedicated HV capacitive voltage divider suitable for 132 kV networks, the design of this device is beyond the scope of this paper.

Future work will aim at performing additional MVT module thermal tests and long-term stability tests. Also, the device performance in various scenarios for lightning tests will be investigated.

## Data Availability

All data underpinning this publication are openly available from the University of Strathclyde KnowledgeBase at https://doi.org/10.15129/7c33bdbf-4734-45e0-8074-389638a88fd9.
